# The COVID-19 Mental Health Content Moderation Conundrum

**DOI:** 10.1177/2056305120948186

**Published:** 2020-08-11

**Authors:** Ysabel Gerrard

**Affiliations:** The University of Sheffield, UK

**Keywords:** COVID-19, mental health, social media, content moderation

## Abstract

At the time of writing (mid-May 2020), mental health charities around the world have experienced an unprecedented surge in demand. At the same time, record-high numbers of people are turning to social media to maintain personal connections due to restrictions on physical movement. But organizations like the mental health charity Mind and even the UK Government have expressed concerns about the possible strain on mental health that may come from spending more time online during COVID-19. These concerns are unsurprising, as debates about the link between heavy social media use and mental illness raged long before the pandemic. But our newly heightened reliance on platforms to replace face-to-face communication has created even more pressure for social media companies to heighten their safety measures and protect their most vulnerable users. To develop and enact these changes, social media companies are reliant on their content moderation workforces, but the COVID-19 pandemic has presented them with two related conundrums: (1) recent changes to content moderation workforces means platforms are likely to be less safe than they were before the pandemic and (2) some of the policies designed to make social media platforms safer for people’s mental health are no longer possible to enforce. This Social Media + Society: 2K essay will address these two challenges in depth.

Mental health charities around the world have experienced an unprecedented surge in demand over the past few weeks and months. In the United Kingdom, for example, the Beat Eating Disorders (2020) charity saw a 50% increase in requests for its services since the nation-wide lockdown was first enforced, and calls to mental health charities like SANE and Anxiety UK were up by 200% at the start of May 2020 (Stephens, 2020). In the absence of access to professional support (Campbell, 2020), mental health apps have been downloaded more than 1 million times since the United Kingdom’s lockdown measures began in March 2020 (Chowdhury, 2020). At the same time, record-high numbers of people have turned to social media to maintain personal connections due to restrictions on physical movement (Newton, 2020a). But organizations like Mind (2020) and even the UK Government (GOV.UK, 2020a) have expressed concerns about the possible strain on mental health that may come from spending more time online during COVID-19.

These concerns are unsurprising, as debates about the link between heavy social media use and mental illness raged long before the pandemic. But our newly heightened reliance on platforms to replace face-to-face communication has created even more pressure for social media companies to heighten their safety measures and protect their most vulnerable users. The pandemic has also widened the net of vulnerability: the increase in demand for mental health services could suggest that people without prior conditions are now struggling. We are indeed witnessing what the United Nations has called a “mental health emergency” (Kelly-Linden, 2020).

To develop and enact these changes, social media companies are reliant on their content moderation workforces, but the COVID-19 pandemic has presented a number of unprecedented challenges to their ongoing efforts. Content moderation is largely enforced by humans who spend their shifts reviewing user reports and “soak[ing] up the worst of humanity in order to protect the rest of us” (Chen, 2014, n.p.; see also Roberts, 2019). Social media companies also employ in-house policy teams who are responsible for setting and enforcing the parameters of “acceptable” social media conduct (Gillespie, 2018), like developing the rulebooks moderators use to respond to user reports (Hopkins, 2017), and enforcing in-platform restrictions like limiting the search results for particular hashtags (Gerrard, 2018) or “shadowbanning” users (Myers West, 2018).

In the context of the COVID-19 pandemic, content moderation workforces face two related conundrums, the effects of which are still likely to be felt when/if the pandemic subsides: (1) recent changes to content moderation workforces means platforms are likely to be *less* safe than they were before the pandemic, and (2) some of the policies designed to make social media platforms safer for people’s mental health are no longer possible to enforce. The remainder of this short paper will address these two challenges in depth.

## Furloughing the Front Line of Social Media

In late March 2020, news broke that major social media companies like Facebook, Twitter, and YouTube had sent their human content moderators home “until further notice”: a role that is “often difficult, if not impossible, to do from home” (Matsakis & Martineau, 2020, n.p.). Many platforms are now relying on artificial intelligence (AI) to take down problematic posts, but this was a near-instant problem (Roberts, 2017). For example, *WIRED* reported that links to articles from legitimate news outlets like *The Atlantic* and *BuzzFeed* had been wrongly removed for violating Facebook’s spam rules, which the platform vaguely attributed to a “bug” (Matsakis & Martineau, 2020, n.p.). While the stakes are high for content like mis/disinformation, platforms also cannot afford to inadequately moderate mental health content at a time when so many of their users are at their most vulnerable.

**Figure 1.**
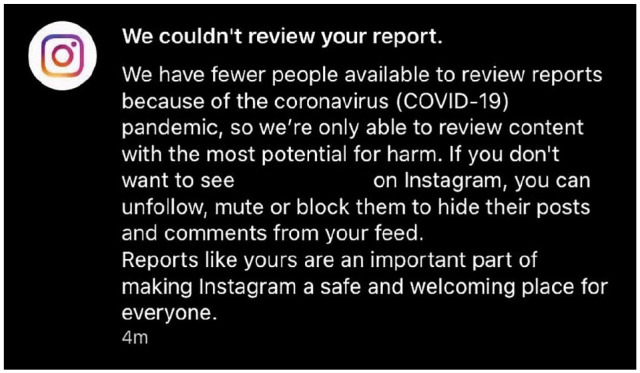
Screengrab of an automated response to a reported Instagram post.

Some social media companies are aware that AI is ill-equipped to moderate mental health-related content (Gerrard, 2018): a moment of transparency praised by the Electronic Frontier Foundation’s (EFF) York and McSherry (2020). But York and McSherry (2020) also warn platforms against relying on AI when the world returns to some version of normal: in their words, “history suggests that protocols adopted in times of crisis often persist when the crisis is over” (n.p.). YouTube has warned users that AI might mistakenly remove videos (YouTube, 2020); Facebook says humans will continue to work on suicide and self-injury prevention (Zuckerberg, 2020, p. 12), and Instagram will still ask humans to review “content with the most potential for harm” (Figure 1).

Much less is known about how platforms like Weibo, WeChat, and VK are handling their content moderation workforces during the pandemic. Western press discourse about non-western platforms tends to focus on the censorship of coronavirus-related content as opposed to changes to their content moderation workforces (BBC News, 2020a): what Newitz (2020) calls “the most important job on the internet.”

But what counts as “content with the most potential for harm”? For example, the eating disorder Anorexia has the highest death rate of any psychiatric condition (Quinn, 2020), but would posts about the promotion of eating disorders—rampant across some newer platforms like TikTok (Gerrard, 2020)—be prioritized according to these new rules? In an article I co-authored with McCosker (McCosker and Gerrard, 2020), we found that people who talk about depression on Instagram largely do so through pseudonymised, humorous meme accounts. Is AI alone capable of reading into these carefully coded contextual cues to detect the necessity of urgent intervention? Sadly, I suspect not.

One of my biggest concerns is that human content moderators struggled to find the time to deal with the onslaught of user reports *before* the pandemic (Roberts, 2017). The UK government has advised people who see “harmful content” on social media to “report it to the site” (GOV.UK, 2020a), and although this seems like the best advice on the surface, it glosses over the workload problems social media giants openly admit they’re facing. If workforces have been reduced *and* we are in a “mental health emergency” (Kelly-Linden, 2020), it’s incredibly unlikely that the remaining moderator workforces at any major social media company will have the time to deal with the current volume of user reports. The consequences of this could be dire.

The COVID-19 pandemic also has implications for the remaining moderators’ own mental health. To ask someone to review the worst content the Internet has to offer during a pandemic is a terrifying, borderline unethical prospect. Roberts’ (2019) decade-long research on content moderation has revealed the prevalence of post-traumatic stress disorder (PTSD) among reviewers; in fact, in May 2020, Facebook agreed to pay a landmark US$52 million “to current and former moderators to compensate them for mental health issues developed on the job” (Newton, 2020b). Although mental health content moderation was far from “complete” (which, I would argue, it never could be), its effectiveness has sadly declined at a time when it is most necessary.

## Widening the Net of Vulnerability

The second, related conundrum social media platforms face is the undoing of their previous mental health content moderation policies, some of which are now dated and others simply unfeasible. Before the pandemic, some globally dominant platforms like Instagram, Pinterest, and TikTok expanded their safety efforts by teaming with independent experts. Instagram, for example, has a Suicide and Self-Injury (SSI) Advisory Board (Facebook, 2020),^1^ and Pinterest has teamed up with experts to design a set of well-being exercises for users who search for self-injury-related terms: “When a pinner enters a related search term, the site will surface a prompt for these exercises” (Pardes, 2019, n.p.). Governments, activists, health professionals, journalists, academics, and other public figures are placing increased pressure on social media companies to minimize the risk of harm that might befall their most vulnerable users. Noteworthy examples from the United Kingdom include the Online Harms White Paper, which takes the first step in developing a new regulatory framework for online safety and “make clear companies’ responsibilities to keep UK users, particularly children, safer online” (GOV.UK, 2020b, n.p.). The tragic suicide of British teenager Molly Russell in 2017 led to another wave of policy alterations at major platforms, mainly Instagram, and which included the introduction of “sensitivity screens” to warn users a post contains sensitive content (Hern, 2019).

While my argument is not that these efforts have been undermined, as the principle of minimizing the risk of online harms extends beyond the pandemic, my point is that the *moments of intervention* have temporarily changed. For example, policymakers across numerous platforms—Instagram, Pinterest, TikTok, Tumblr, to name a few—have long chased harmful hashtags and restricted users’ access to them. But Chancellor et al. (2016) found that users develop code words to work around bans. As an example, the tag #proana (a portmanteau term to denote the promotion of anorexia) might become #proanaaa. A lot of work goes into identifying these terms and then chasing down related tags, but I worry that the code words will have changed in tandem with people’s mental health experiences. As more content moderation centers re-open (BBC News, 2020b), it is important to acknowledge that some of the original actions moderators took will no longer work, and policymakers likely don’t have enough information about the link between COVID-19 and mental health to adapt accordingly (and quickly).

Suicide prevention efforts represent another change to moments of intervention. Since 2017, Facebook has contacted first responders to conduct “wellness checks” on people who the platform’s AI systems and human moderators identify as being at imminent risk of suicide (Zuckerberg, 2018). This particular practice has faced intense criticism, including concerns about the risk of false positives (people who are wrongly identified as being suicidal) and the consequences of such an error, which include Facebook users undergoing unnecessary psychiatric evaluation (Thielking, 2019). But this practice—rightly or wrongly implemented by the platform—is simply unfeasible in the current climate, as first responders in most places around the world are overwhelmed.

Social media companies already had a long way to go in their efforts to protect their users: should healed self-harm scars be censored? What should happen to “borderline” content (posts that don’t quite break the rules but sound alarm bells anyway)? How long should a suicide note stay up for? How can moderators be sure a post “promotes” an eating disorder? But the goal posts have shifted. The work that goes into answering these questions—including qualitative and quantitative information about people’s experiences of mental health conditions—is no longer entirely applicable.

## Social Media: A Psychiatrist’s Biggest Ally?

The word “unprecedented” is ubiquitous in the current climate, and for good reason. This is indeed an unprecedented situation and we don’t yet know how it will affect people’s mental health. What we do know with certainly is that we will feel the repercussions of alterations to social media’s content moderation workforce for years to come. For perhaps the first time, the reduction to human content moderation has vividly brought to light “the traces, which are so often hidden, of human intervention” (cited in Matsakis & Martineau, 2020, n.p.). These traces include errors and blind spots, and once again remind us how impossibly traumatic this job is for the human content moderator workforce. The COVID-19 pandemic also renews debates about platforms’ parameters of responsibility: where does their responsibility for users’ mental health start and end? Who should be responsible for overseeing their interventions?

Chaudhary and Vasan (2020) believe that technology and social media companies are “uniquely suited to be a psychiatrist’s biggest ally in our mission to improve mental health for the 2 billion people around the world struggling with brain and behavioral health disorders” (n.p.). I mostly agree, and in the throes and aftermath of the COVID-19 pandemic, researchers, medical professionals, and tech company workers need to commit to working together, sharing resources, and possessing a genuine, moral desire to help social media platforms’ increasingly vulnerable global userbase.

## Notes

1. I have been a member of Facebook and Instagram’s Suicide and Self-Injury (SSI) Advisory Board since March 2019. At present, this is an unpaid position and I predominantly advise on Facebook and Instagram’s policies about eating disorder content.
